# In Situ Characterization of Polycaprolactone Fiber Response to Quasi-Static Tensile Loading in Scanning Electron Microscopy

**DOI:** 10.3390/polym13132090

**Published:** 2021-06-24

**Authors:** Alexander Delp, Alexander Becker, Daniel Hülsbusch, Ronja Scholz, Marc Müller, Birgit Glasmacher, Frank Walther

**Affiliations:** 1 Department of Materials Test Engineering (WPT), TU Dortmund University, 44227 Dortmund, Germany; daniel.huelsbusch@tu-dortmund.de (D.H.); ronja.scholz@tu-dortmund.de (R.S.); frank.walther@tu-dortmund.de (F.W.); 2 Institute for Multiphase Processes, Leibniz University Hannover, 30823 Garbsen, Germany; mueller@imp.uni-hannover.de (M.M.); glasmacher@imp.uni-hannover.de (B.G.); 3 Lower Saxony Center for Biomedical Engineering, Implant Research and Development (NIFE), 30625 Hannover, Germany

**Keywords:** in situ tensile testing, scanning electron microscopy, microstructure, damage mechanisms, tissue engineering, electrospinning, fiber orientation, polycaprolactone

## Abstract

Microstructural responses to the mechanical load of polymers used in tissue engineering is notably important for qualification at in vivo testing, although insufficiently studied, especially regarding promising polycaprolactone (PCL). For further investigations, electrospun PCL scaffolds with different degrees of fiber alignment were produced, using two discrete relative drum collector velocities. Development and preparation of an adjusted sample geometry enabled in situ tensile testing in scanning electron microscopy. By analyzing the microstructure and the use of selected tracking techniques, it was possible to visualize and quantify fiber/fiber area displacements as well as local fractures of single PCL fibers, considering quasi-static tensile load and fiber alignment. The possibility of displacement determination using in situ scanning electron microscopy techniques for testing fibrous PCL scaffolds was introduced and quantified.

## 1. Introduction

**Figure 1:**
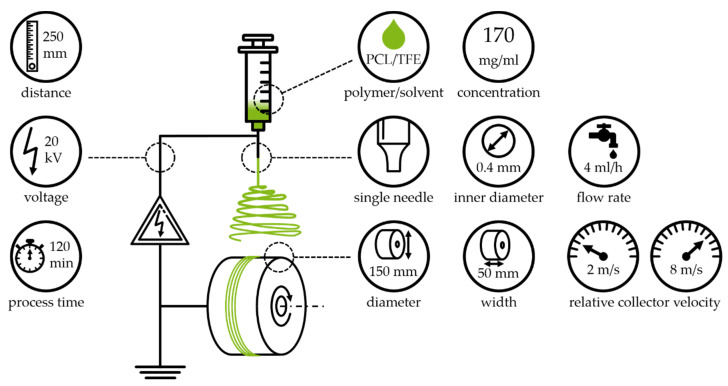
Electrospinning device with process parameters and settings used in this study. The depicted system consists of a polymer processing unit (syringe, blunt cannula, polyethylene tubing and syringe pump), a high voltage supply and a collector unit (rotating drum collector and electric motor). The adjusted parameters are displayed: polymer and solvent, concentration, needle setup and dimensions, flow rate, drum collector dimensions, relative collector velocity, tip to collector distance, voltage and overall process time.

In the context of modern medicine, transplantations and implantations are a fundamental part of advanced therapy. This leads to new challenges, such as the increasing demand and the following shortages of donor organs [1,2]. So-called tissue engineering (TE) aims towards the recreation of tissues and ultimately solid organs, as a possible solution for these challenges. A major aspect of TE is the recreation of the extracellular matrix (ECM), which presents different micro- and macroscopic fiber structures [3,4,5]. With this in mind, polymers are commonly used materials in TE [6,7,8]. In fact, the fabrication of fiber scaffolds by electrospinning (see Figure 1) has produced great results for several different combinations of polymers and solvents (e.g., polycaprolactone (PCL) in trifluoroethanol (TFE) or polyethylene oxide in water) [9,10,11]. The resulting fibers inherit diameters between a few hundred nanometers and a few microns [12,13]. In order to recreate complex tissues, such as tendon–bone junctions, scaffolds with diversified fiber diameters and alignments are necessary [14,15,16]. The intended application of these scaffolds, e.g., the treatment of chronic tears in the rotator cuff or as a drug delivery system, calls for a thorough characterization of the resulting properties [17]. The focus of this study lies in the behavior of electrospun PCL fiber scaffolds under mechanical loading. There is no standard for the mechanical testing of PCL; therefore, different sample geometries and preparation techniques are used. Furthermore, the fibrous nature of electrospun PCL results in deviation of the true cross section when determined with conventional thickness measurements, and therefore hampers the comparability of recent results [18]. Sowmya et al. (2021) recently published a review of PCL applications for tissue engineering [19].

Microstructural changes of polymer structures can be analyzed reliably using (in situ) computed tomography and scanning electron microscopy (SEM) [20,21]. Early in situ experiments using tensile testing and a polarizing microscope were conducted by H. Ambron (1892) with a stretching instrument for gelatin [22]. A device for the in situ testing of single fibers in SEM and first results were introduced in 1989 [23]. In addition to adjusted setups and machines for testing single fibers in the form of textiles such as polymer fiber laying inside SEM, a tailored sample geometry is expedient. The testing of PCL is not standardized; therefore, Zernetsch (2016) developed a testing method based on polymer standard ISO 527 for testing PCL fiber scaffolds [14,24,25]. An extraction scheme for strip tensile samples from fabrics is included in ISO 13934-1 appendix B, which is relevant for the manufacturing of PCL samples. Withdrawn DIN 53816 mentions a paperboard frame for brittle textile fiber tensile samples. The testing of fiber reinforcement of ceramic fiber composites is standardized within EN 1007-4 and contains a paperboard-reinforced sample type. For testing carbon monofibers, a similar geometry is described with ISO 11566. Kumari et al. (2010) successfully used analogous sample geometry for the tensile testing of carbon monofibers and multifibers in SEM [26]. When testing cottonid samples via in situ SEM methodology, it was shown that local charging of a non-conductive, carbon-coated sample can hint to occurring damages in fiber materials. On the other hand, it impairs SEM imaging. Furthermore, polymers are vulnerable to creep and relaxation depending on load mode, i.e., constant strain or constant stress, at intermittent in situ testing [27,28].

In this study, samples were prepared from electrospun PCL fiber scaffolds and investigated with an advanced in situ tensile testing methodology inside an SEM device. The results of the uniaxial tensile testing were analyzed and compared to the microstructure. Differences in strain development and damage development in single fibers were shown.

## 2. Materials and Methods

### 2.1. Electrospun PCL Samples

#### 2.1.1. Electrospinning

Figure 1 depicts the general setup of an electrospinning device. It generally consists of a grounded collector, a high voltage supply and an emitter. The polymeric solution enters the electrical field vertically (top-down electrospinning) through the emitter [11,14]. Due to the fluids’ surface tension, the polymeric solution forms a droplet. As soon as the surface tension is overcome by the applied forces, induced by gravity and the electrical field, a fiber jet is emitted and accelerated towards the collector. The fiber diameter is constantly decreasing, due to solvent evaporation and stretching, until deposition on the collector. The properties of the fabricated fiber scaffolds depend to a high degree on solution, process and ambient parameters, as well as the setup orientation [11,24,29]. The relative velocity, at the surface of the collector, is of special interest for this study. As shown by Fricke et al. (2019), an increase in relative collector velocity leads to an increased degree of fiber orientation [9,14].

#### 2.1.2. Processing System

The used electrospinning device (see Figure 1) was assembled according to Fricke et al. (2019) and is composed of a syringe pump, a syringe, polyethylene tubing, a blunt cannula, and an electric motor driving the rotating drum collector [6,9,14].

#### 2.1.3. Experimental Procedure and Parameter Settings

The samples were manufactured out of a solution of polycaprolactone (PCL, 80 kDa, Sigma-Aldrich Chemistry Corporate, St. Louis, MO, US) in 2,2,2-TFE (99.8%, abcr GmbH, Karlsruhe, Germany) with a concentration of 17% (*w*/*v*) (170 mg/mL). The solution was prepared according to Fricke et al. (2019) [9,14]. The samples were fabricated with a 250 mm tip-to-collector distance, voltage of 20 kV, relative collector velocities of 2 m/s and 8 m/s, and a process duration of 120 min (Figure 1).

#### 2.1.4. SEM-Based Analysis of Fiber Diameter and Degree of Orientation

Fiber diameter and the degree of orientation were determined based on ten SEM (S-3400 N, Hitachi High-Tech Analytical Science Ltd., Tubney Woods, Abington, UK) images: five for each of the two relative collector velocities. Afterwards, the images were analyzed using image analysis software (AxioVision^®^, Carl Zeiss AG, Jena, Germany) as described by Fricke et al. (2019) [14,30].

### 2.2. In Situ Tensile Testing of Polymer Fibers

#### 2.2.1. Test Setup for In Situ Testing

**Figure 2:**
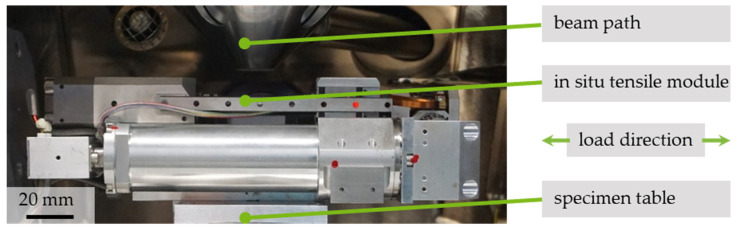
Mounted in situ tensile testing device for SEM.

For the study, the SEM (Mira 3 XMU, Tescan, Brno, Czech Republic) apparatus was equipped with a micro tension/compression module with a load capacity of 200 N (micro-tensile compression tester, Kammrath & Weiss GmbH, Dortmund, Germany) as shown in Figure 2. The clamping device consisted of two roughened blocks with a screw fixing. In situ as well as quasi-in situ tensile testing was conducted by choosing a high scanning rate with fixed magnification in addition to video recording stopping the test at discrete strain-values with higher SEM scanning rates and variable magnification.

#### 2.2.2. Sample Preparation

**Figure 3:**
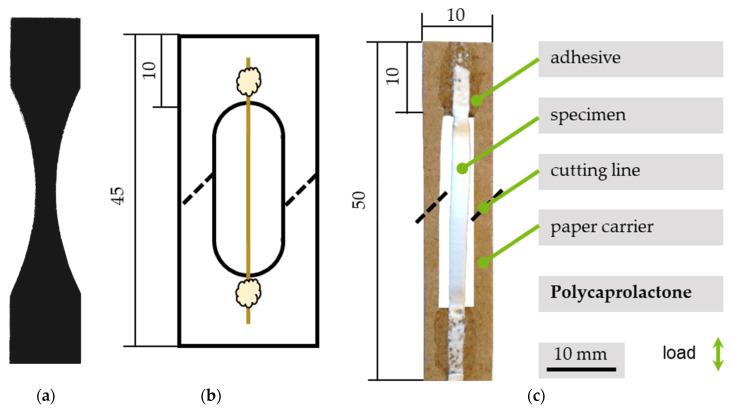
Ideal sample geometry for the in situ device as a basic condition for sample development (**a**). Standardized sample geometry for testing single carbon filaments according to ISO 11566 as a basis for scaffold attachment (**b**) and the resulting geometry for testing PCL fiber samples with in situ SEM methodology (**c**).

The typical sample geometry for in situ tensile testing has a centric rejuvenation for crack initiation in a controlled area, as shown in Figure 3a. Combined with a sample type for testing monofibers according to ISO 11566 (Figure 3b), the geometry was adjusted, as shown in Figure 3c. Previous investigations, for samples obtained with the same device and set of parameters, showed homogeneous fiber deposition on the collector for up to 75% of fiber mat width. These results were considered for the following preparation method and sample geometry [31]. For preparation, rectangular stripes in two orientations and to the rotational direction of the drum collector (0°, 90°, respectively) were manually cut out of the electrospun PCL scaffolds using a scalpel. The samples were glued onto a protective frame using a cyanoacrylate-based adhesive (UHU Blitzschnell mini, UHU GmbH & Co. KG, Buehl, Germany). Rectangular paperboard frames (with exterior dimensions length, l = 50 mm; width, w = 10 mm) and a centrically arranged inner clipping of l = 30 mm, w = 6 mm were used to adopt an appropriate geometry and ensuring a load-free clamping. A drop of adhesive was placed in the middle of both short sides of the paperboard for assembly. After removing the aluminium foil, used for segregation between the PCL and drum collector, with tweezers, the ends of the samples were placed one after another on the adhesive without applying longitudinal stress.

Shortly before testing and after a curing time of at least 24 h, the sample underwent carbon coating (Cressington Carbon Coater CR 208 carbon, Tescan, Brno, Czech Republic). This step is crucial in order to ensure conductivity and minimize local charging because PCL is not conductive.

### 2.3. Qualitative and Quantitative Evaluation of Microscopic Images

Different methods were used to describe the fiber movement due to quasi-static loading of the PCL scaffolds. First, the SEM results were aligned with reference to recognizable structures for each series, marked as reference points. Characteristic structures were selected for each imaging series and quantitatively evaluated by use of image processing software Image J (V.1.53e, Wayne Rasband, National Institute of Health, Bethesda, MD, USA). The presented application of three different graphical techniques allows a qualitatively comparison: vectors of different colors are used to illustrate the fiber movement and form visible displacement fields. For the fast comparison of structural displacements, fibers in a defined strain condition were schematized and overlaid with microstructural images in a different strain condition. The third method was the use of a grid pattern in combination with vectors as well as schematized structure overlays, which enabled the description of local fiber movement.

### 2.4. Statistical Analysis

If not stated otherwise, the results for the determination of fiber diameter and degree of orientation are depicted as boxplots with outliers. The interquartile range (IQR) for all boxplots represents 50% of the measured data and is illustrated as a closed box. The mean value is depicted as a rhombus, while the median value is shown as horizontal line within the IQR. The displayed whiskers are defined as 1.5 times the IQR, and the distance between those whiskers represents the dispersion of the values. All values located outside of the 1.5 × IQR are individually displayed as dots. These were defined as outliers, with regard to the IQR, but do not necessarily have to be considered as extreme values [9].

In order to evaluate the application of parametric or non-parametric tests, quantile–quantile plots (QQ Plots) were generated. In case of the indicated parametric tests, a two-samples *t*-test was conducted, to investigate possible differences between the groups [32]. For indicated non-parametric tests, the Mann–Whitney test was carried out [33]. Differences were considered significant at *p* < 0.05 (*), *p* < 0.01 (**) and *p* < 0.001 (***). All data were analyzed using statistical analysis software (Origin 2018b, OriginLab Corporation, Northampton, MA, USA) [9,14].

## 3. Results

### 3.1. Microstructure of PCL

**Figure 4:**
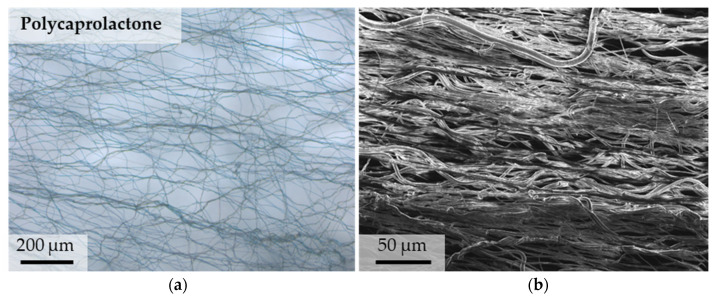
Light microscopic image of manually stretched PCL shows corrugated and straight single fibers (**a**) compared to an SEM image of aligned PCL with visible fiber alignment and higher depth of focus but loss of color information (**b**).

In addition to a higher resolution, SEM recordings provide a higher depth of focus in comparison to light microscopy images. Disadvantages are the loss of information regarding color as well as a required sputtering of samples. In Figure 4a, a light microscopy image of manually stretched PCL is compared with an SEM recording (Figure 4b). It can be seen that part of the fibers are aligned, and another part shows a corrugated structure. Furthermore, the fibers show differences in diameter.

### 3.2. SEM-Based Analysis of Fiber Diameter and Degree of Orientation

**Figure 5:**
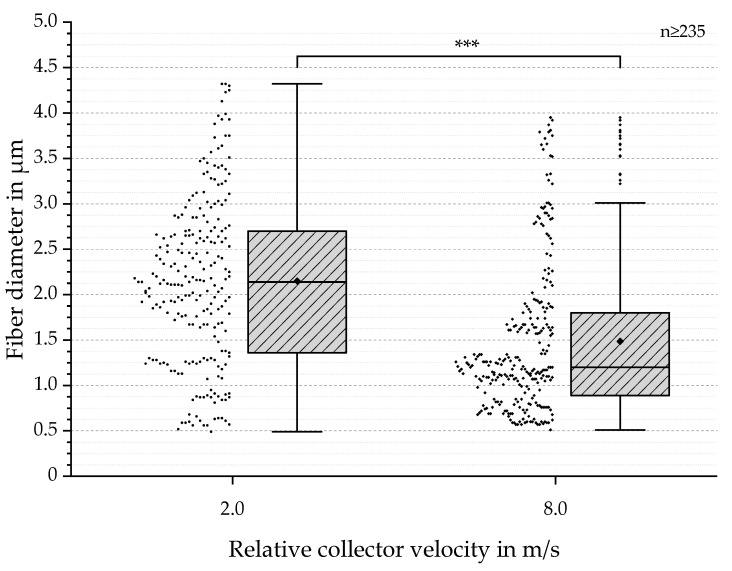
Boxplots with outliers of fiber diameters in µm for relative collector velocities of 2.0 and 8.0 m/s. In addition, individual values are presented as dots on the left side of each boxplot. The datasets show a mean of 2.1 µm for 2.0 m/s and 1.5 µm for 8.0 m/s. Furthermore, the comparison shows a decreased IQR and dispersion for increased relative collector velocity. Based on the QQ Plots, the Mann–Whitney test was conducted and resulted in highly significant differences between the two groups: *** (*p* < 0.001).

SEM-based analysis of fiber diameter resulted in diameters between 0.5 and 4.3 µm for 2.0 m/s and a calculated mean value of 2.1 µm; correspondingly, for 8.0 m/s, the calculated mean value was 1.5 µm, with a range of 0.5 to 4.0 µm (see Figure 5). Despite these similar findings, the observed dispersion and IQR decreased with increasing the relative collector velocity, from 1.3 (2.0 m/s) to 0.9 (8.0 m/s). These results are consistent with findings by Fricke et al. (2019, 2020) [9,14]. Due to the results of the conducted QQ Plots, the Mann–Whitney test was applied. Highly significant mean differences were found (*** *p*-value < 0.001) (see Figure 5).

**Figure 6:**
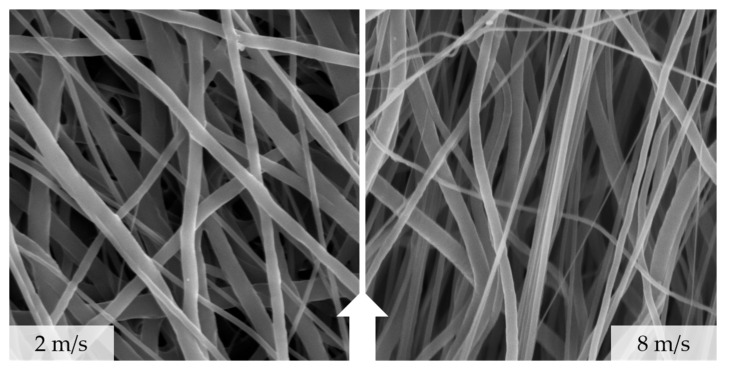
Alignment of fibers according to the relative collector velocity of 2 m/s (**left**) vs. 8 m/s (**right**); the white arrow indicates the rotational direction.

**Figure 7:**
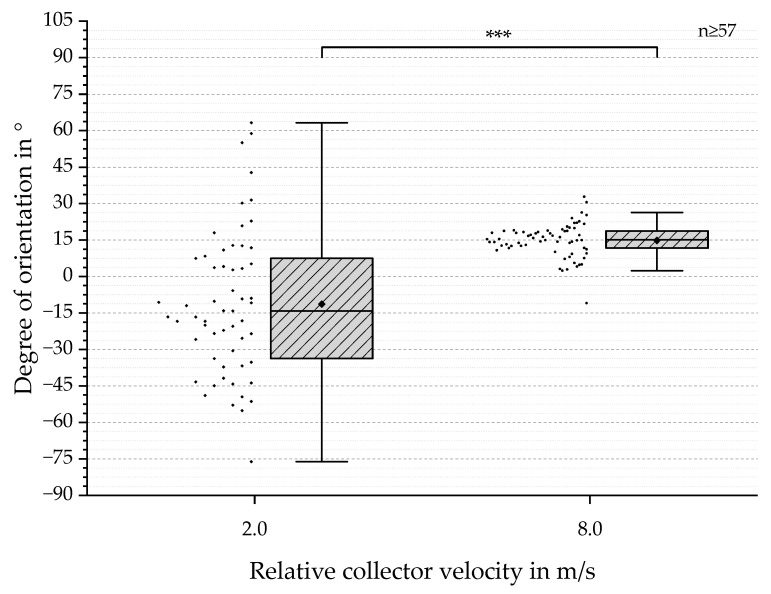
Boxplots with outliers for the degree of orientation in degrees for relative collector velocities of 2.0 and 8.0 m/s. Additionally, individual values are presented as dots on the left side of each boxplot. For 2.0 m/s, the majority of values were located around −11°, whereas the values for 8.0 m/s were mainly distributed around 14°. A wider distribution for 2.0 m/s can be observed and the comparison of both datasets shows a decreased IQR and dispersion for increased relative collector velocity. Due to the QQ Plots indicating normal distribution and the values being independent, a two-samples t-test was conducted. Statistically significant mean differences were found and labeled as follows: *** (*p* < 0.001).

In order to evaluate the degree of orientation, SEM-based analyses were performed (see Figure 6). The results ranged from −76.1° to 63.2° for 2.0 m/s and from −10.9° to 32.8° for 8.0 m/s. The mean values were calculated as −14.2° (2.0 m/s) and 15.2° (8.0 m/s). The displayed IQR and dispersion showed a major decrease with increasing the relative collector velocity (see Figure 7), similar to the results for fiber diameter measurements (see Figure 6) [9,14]. Based on the QQ Plots, a two-samples *t*-test was carried out, which revealed highly significant differences between the two groups (*** *p*-value < 0.001) (Figure 7).

### 3.3. Transversal Contraction under Tensile Load

**Figure 8:**
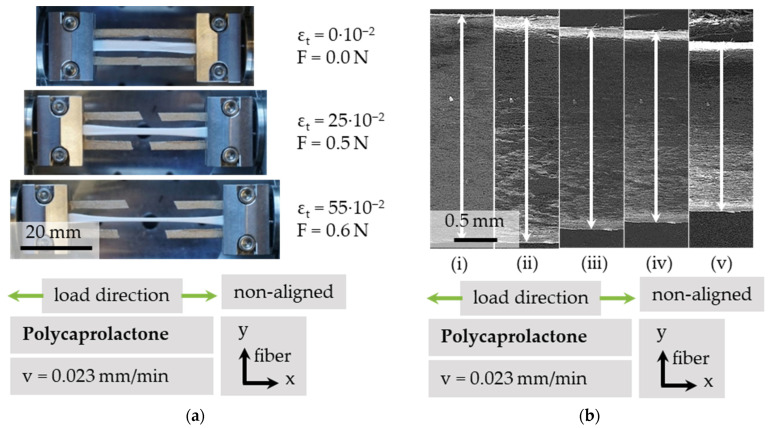
Tensile testing of customized PCL sample in macroscopic view (**a**) and resulting transversal contraction of non-aligned PCL, monitored via SEM (**b**). The corresponding load steps are shown in Table 1.

The load was evenly applied from both sides, resulting in a consistent strain. The lateral contraction orthogonal to load direction ε_cy_ and total nominal strain in load direction ε_t_ were calculated by dividing the change in length Δl = l − l_0_ by the initial gauge length l_0_. Figure 8a shows three stages of a tensile test: First, at the initial condition, unloaded with cut frame. Secondly, at a nominal total strain of ε_t_ = 25 × 10^−2^ with a load of F = 0.5 N. At a strain of ε_t_ = 55 × 10^−2^ and a load F = 0.6 N, lateral contraction was already distinctly visible on a macroscopic scale. When observed at a microscopic scale, as shown in Figure 8b for non-aligned PCL, in this example, a strain of ε_t_ = 3 × 10^−2^ resulted in a lateral contraction ε_cy_ = −11 × 10^−2^ and a force of F = 0.1 N. At a strain of ε_t_ = 10 × 10^−2^, lateral contraction reached ε_cy_ = −32 × 10^−2^ and force increased up to F = 1.8 N, as shown in Table 1. The lateral contraction ε_cy_ of a fiber scaffold can reach up to 3.8 times its longitudinal strain ε_t_. There is a possibility of an unknown bulging influence; therefore, the authors denote the lateral contracture as contraction and not as true strain ε_t_.

### 3.4. Response of Microstructure Due to In Situ Tensile Testing

#### 3.4.1. Stress–Strain Behavior of PCL under Tensile Loading

**Figure 9:**
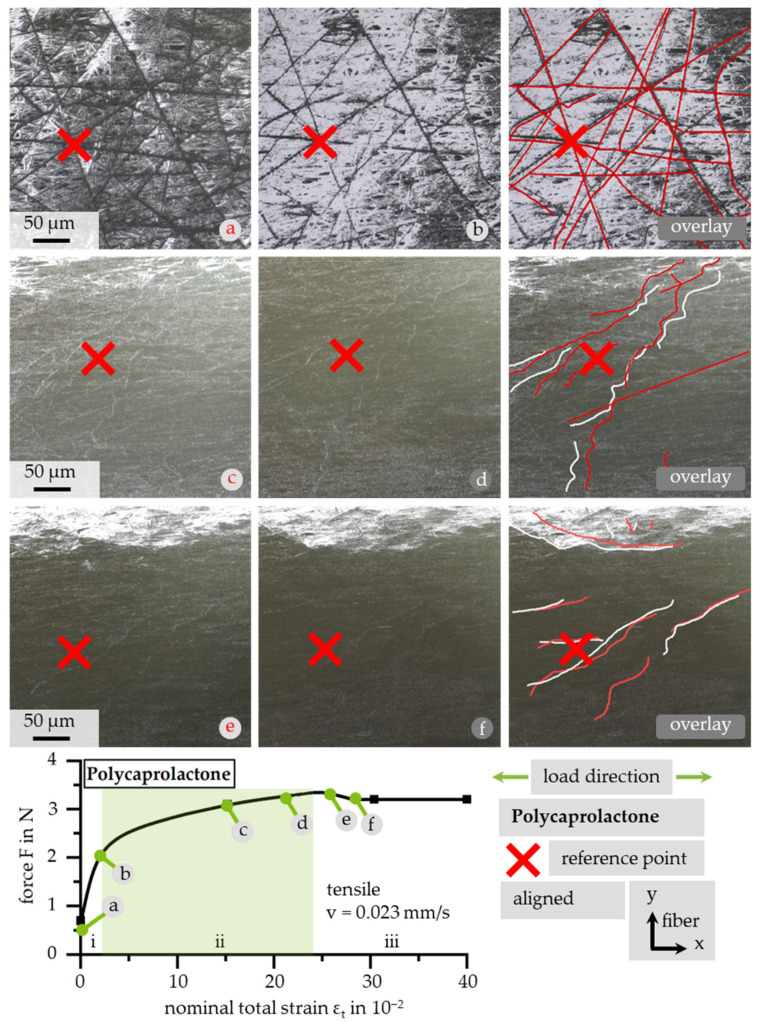
Definition of three nominal total strain sections with reference to an exemplary force—nominal total strain curve. Development of fiber movement and alignment due to tensile load with overlay illustration of distinctive fibers and the mapping of conditions along the force–strain curve. The superimposed image is denoted by the used overlay color.

Due to the lower scatter in fiber diameter, for microstructural investigations, samples of aligned PCL were used. Furthermore, the scatter in fiber orientation resulting from low relative collector velocities in combination with a limited view field of SEM led to the need for manual readjustments when recording. As visualized in Figure 9, the force–strain curve of PCL can be categorized in three sections: (i) viscoelastic strain; (ii) elastoplastic strain with increasing force; and (iii) increasing strain with no increase in force. Furthermore, the conditions of fibers at discrete loading points are depicted. Each recording corresponds to the marked point in the force–strain curve. In addition to recordings of the microstructure, traced overlays of noticeable fibers in two conditions are shown. The basis is marked with the corresponding letter from the force–strain curve, colored in black/white. The overlay is depicted in red. As a reference, a red marker is positioned at the adjustment point for picture overlay. Due to the immense elongation and limited view field, the initial condition is not recognizable after applying large strains. Additionally, a reduction in imaging quality is noted due to local charging on the sample’s surface. In the first area, the viscoelastic strain region, displacements within the whole fiber area are recognizable (a–b). With further deformation, single fibers move non-consistently with respect to the whole area (c–d). In section iii, a straightening of corrugated fibers can be noticed (e–f).

#### 3.4.2. Quantitative Analysis of Fiber Movement

**Figure 10:**
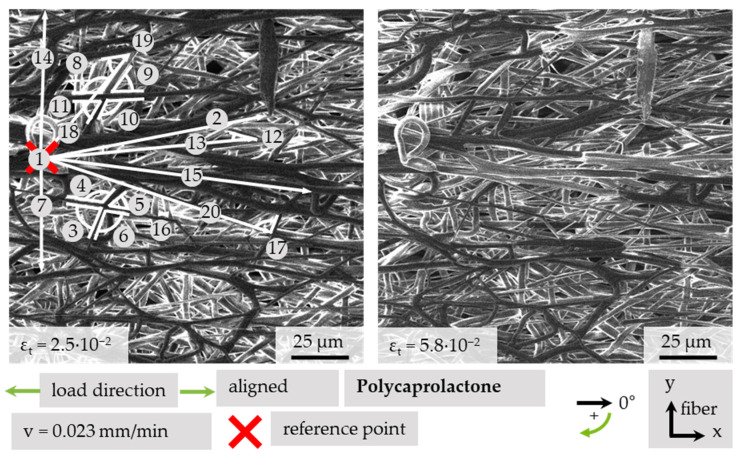
Measurement of fiber movement of PCL due to tensile testing.

To characterize and visualize the movement of fibers quantitatively, it is possible to measure angles and distances via imaging processing software. It is necessary to have a reference scale to qualify the data. A measurement as just described was conducted at two conditions on an aligned sample at ε_t_ = 2.5 × 10^−2^ and ε_t_ = 5.8 × 10^−2^, as shown in Figure 10. The measured values are shown in Table 2. Two types of angles are considered. If a length is given on the left, the angle corresponds to the global coordinate system. Other cases are marked with *. Those angles are measured between two defined fibers.

The movement of the structure under tensile loading has been quantified by measuring the distances between defined, distinctly recognizable structure elements and a fixed reference point. Additionally, the fiber angle can provide more information about the direction of movement. The structure expands under tensile load in the load direction, as shown with measurement points 2, 15 and 20. A lateral contraction can also be monitored by observing the angle. The local strain in the projected x-direction differed from the total strain, because the local projected strains are ε_lx,2_ = 2.7 × 10^−2^, ε_lx,15_ = 3.0 × 10^−2^, ε_lx,20_ = 1.8 × 10^−2^ at a total strain difference in the tensile direction of Δε_tx_ = 2.3 × 10^−2^. A great difference was found with points 13 and 18, which seem to lie at a level beyond the reference point. Here, the strain is ε_lx,13_ = −10.5 × 10^−2^, because the whole structure has moved towards the reference point. Points 7 and 14 show local lateral contraction regarding the reference point with ε_ly,7_ = −9.3 × 10^−2^ and ε_ly,14_ = −8.3 × 10^−2^. This finding coincides with the relationships shown in Table 1. The angles of measurement points 8–11 are between two non-linked fibers. The angles shown with numbers 3–6 are connected because of a coalesced fiber crossing. Due to differences within a single fiber diameter, the four angles of a fiber crossing do not necessarily add to 360 degrees. The angles denoted with 3 and 5 are mostly orientated in the x-direction. Therefore, they decrease with increasing positive strain, whereas the values of y-orientated angles 4 and 6 increase with increasing strain. The non-linked angle quartet, consisting of points 8 to 11, also show this behavior depending on the angle opening direction, but due to missing connection, the value of point 10 is not affected by the change of the other angles. Furthermore, changes in single angles between fibers can be seen with points 12, 17, and 19.

#### 3.4.3. Qualitative Analysis of Fiber Movement

**Figure 11:**
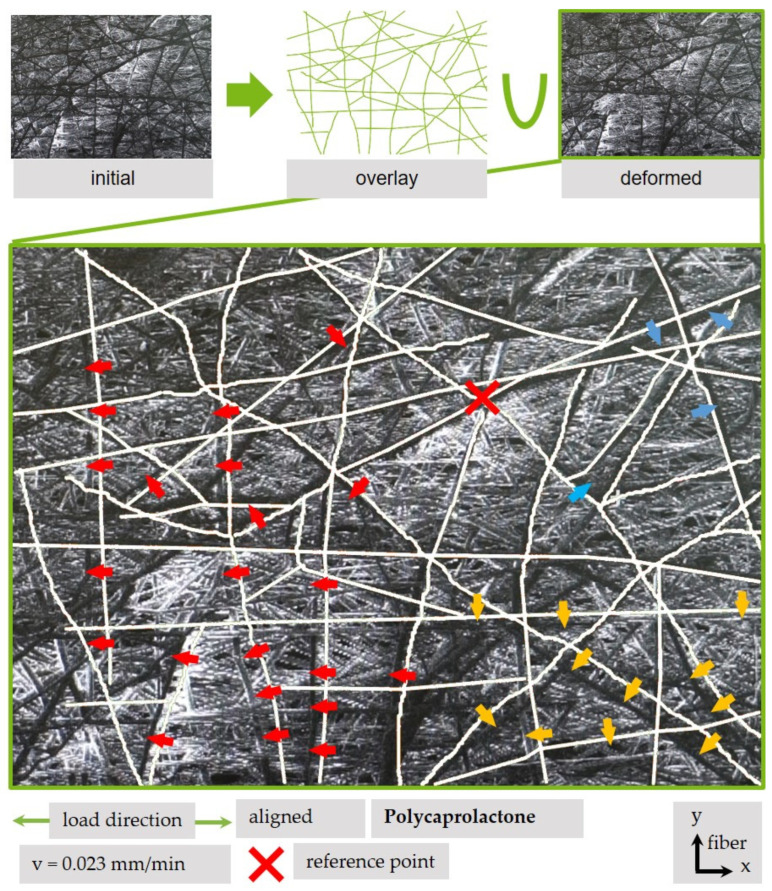
Indicators and schematic overlay for the movement of aligned PCL fibers under tensile load.

In addition, a qualitative analysis can be helpful to monitor tendencies in the movement of a fiber structure. In Figure 11, a deformed condition of a fiber scaffold is superimposed by a redrawn structure of characteristic and recognizable fibers in a condition with lower strain. By using the overlay, it is possible to identify the movement direction of chosen fibers on the surface. The strain field can be described using a vector field. As expected, the majority of fibers move in tensile direction (red arrow). However, some areas move in different directions; for example, the fiber area on the lower-right side (yellow) and on the upper-right side (blue).

**Figure 12:**
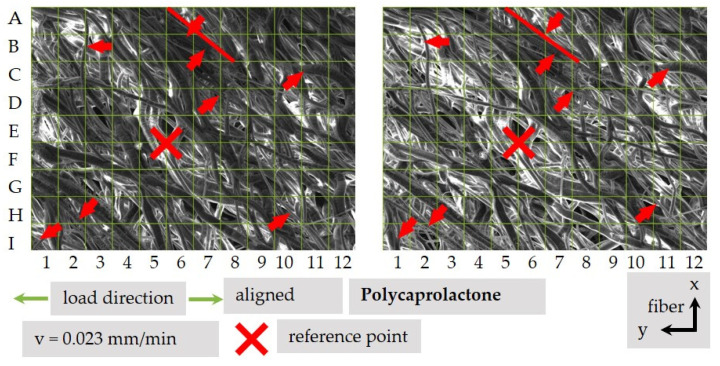
Fiber movement of a 90° sample.

A further possibility to evaluate the structure and fiber movement is to divide the area into a grid pattern (see Figure 12). It becomes clear that in addition to the movement of single fibers, a movement of fiber bundles in an angle of 90° regarding their orientation direction is possible for, e.g., fields I1, B3, D7 in tensile direction, if the sample is cut in a 90° orientation. This is shown with a red line in area A6–C8.

#### 3.4.4. Local Fiber Fracture

**Figure 13:**
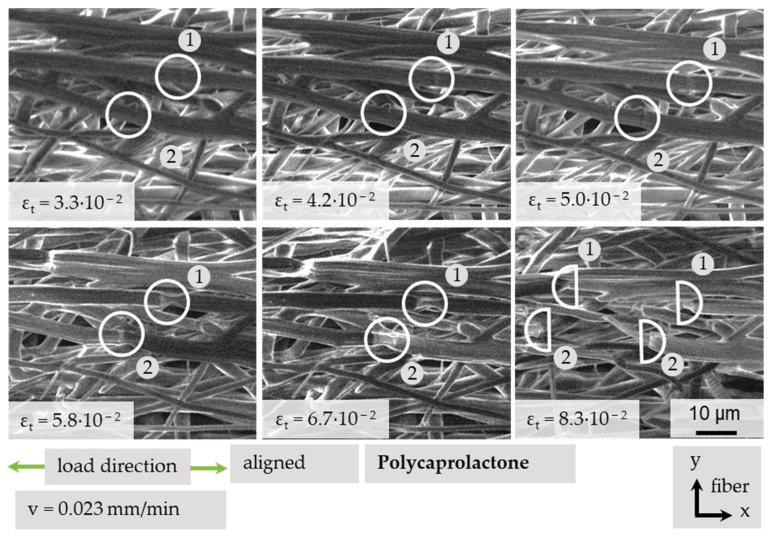
Local necking and fracture of single PCL fibers under tensile load.

After introducing and using different methods to describe the movement of single fibers, fiber bundles and the complete structure, an evaluation of microstructural changes regarding the damage mechanisms of fibers is necessary. It was possible to record necking and the fracture of different fibers under tensile load with the in situ SEM methodology as shown in Figure 13. Under increasing strain, initially unremarkable areas (ε_t_ = 3.3 × 10^−2^) develop local changes (ε_t_ = 4.2 × 10^−2^; (1) and (ε_t_ = 5 × 10^−2^; (2). As can be seen here, this does not necessarily happen for all fibers at the same strain values. Further increase in strain leads to local necking at the conspicuous fiber changes (ε_t_ = 5.8 × 10^−2^; (1) (ε_t_ = 6.7 × 10^−2^; (2)), and finally, to single fiber fracture.

## 4. Discussion

The degree of orientation shown in Section 3.2 (Figure 7) depicts a similar trend (IQR and dispersion) compared to that described by Fricke et al. (2019; 2020). At the same time, the calculated mean values of −14.2° and 15.2° differ from the expected 0° [9,14]. This could be explained by an inhomogeneous electrical field distribution, thus leading to an offset during fiber deposition on the collector. Another explanation can be found in the SEM-based analysis protocol, previously described by Fricke et al. [14]. In order to evaluate the degree of orientation, the rotational direction has to be marked. This happens by folding the sample in the direction of the relative collector velocity. Afterwards, this fold mark is superimposed by a line during the image analysis. Therefore, an angular deviation of this line would have an impact on the results. A possible solution would be the use of an alternative method [9,14].

Subsequently, significant structural changes caused by the tensile loading of PCL fiber mats were identified: first, increases and decreases, respectively; secondly, in angle values between single fibers; thirdly, crossings of non-linked fibers and between linked fibers; finally, changes in the distance among single fibers as well as local changes in elongation, which may display higher strain values as recognizable on a global scale. It is of further importance to analyze the orientation of single fibers, between which the angle is measured. If the fiber angle, the tensile direction, and the orientation of fibers is known, a profound characterization of the fiber structure is possible. It is sufficient to use a 180° angle scale to characterize the dislocation of a single sample fiber orientation, because the vector direction depends on the chosen local reference point. The orientation of material or fiber, as well as the number of linkages, determines which quantity of fibers responds in which way. This means that the structure of the material is decisive with respect to the development of local areas, which act differently under the same global strain. This is an important observation regarding the alignment. The stress–strain curve can globally be divided into the abovementioned three sections, but fractures of single fibers can already occur in the second section. The results suggest that after a viscoelastic stretching, first, fibers react with local plastic deformation and necking. Second, after primary segregations, the stress is transferred to corrugated fibers and linked fiber bundles. With further elongation, more connections and fibers break. Corrugated fibers align until fracture.

These aspects considered, future studies should focus on an appropriate sample preparation protocol to ensure reproducibility, comparability and reliability of the results.

A difficulty in using in situ SEM methodology for the characterization of PCL is the minor conductivity in initial condition and a damage development in used sputter coatings due to immense strain values. A fragmentation of the brittle carbon layer and, as a result, a diminution of the electric conductivity is presumed. This causes a reduction in imaging quality. In addition to the sputter coating, the functional coating of electrospun PCL for enhanced biocompatibility and fiber conductivity is the aim of recent research [34]. For the physiological qualification of PCL, the viscoelastic area until ε_t_ = 3 × 10^−2^ − 5 × 10^−2^ is of further importance. In this area, the image quality is sufficient. A defined endowment of the material could be helpful to track movements using digital image correlation software. The accuracy of manual measurement at a resolution of 2048 × 1536 pixels with 500× magnification and an ideal contrast ratio depends on the pixel grid, the digital zoom factor of the image processing software, the software itself, as well as the manual imprecision. Under ideal circumstances, the accuracy with the used combinations can reach up to +0.110/−0.113 μm or +1.03/−1.06 pixel.

## 5. Conclusions

If the angle between single fibers, the tensile direction, and the orientation of fibers with respect to the rotational direction of the drum collector is known and a reliable scale is provided, a quantitative statement to characterize the fiber movement under load is possible using the in situ SEM methodology. As a feedback regarding the electrospinning parameters, the degree of orientation can be determined through an SEM-based measurement. For a fast assessment of the behavior of PCL under load, qualitative evaluations of fiber movement by using a vector field is possible. An enhanced method is provided by using a chessboard pattern and describing the movement of fibers and fiber areas between two conditions. It is conceivable to use the introduced testing methods for the adjustment of manufacturing parameters to generate defined structural properties and regulate the microstructural responses to external load. The influence of fiber diameter is of prime importance. The mechanical failure of the scaffold under tensile loading as a result of local necking and breakage of single fibers is an important first observation for characterization of the acting damage mechanisms. However, another question is the behavior of fiber linkages. On a nanostructural scale, the alignment of polymer chains due to electrospinning could explain further structural responses. Generally, additional research is needed concerning the fiber orientation as well as the behavior of PCL in physiological condition under in vitro fatigue load.

## Figures and Tables

**Table 1:** Strain and contraction of non-aligned PCL, quantified via SEM and imaging processing software, corresponding to Figure 8b.

No.	Strain, ε_t_	Contraction, ε_cy_	Force, F
i	0 × 10^−2^	0 × 10^−2^	0.0 N
ii	3 × 10^−2^	−11 × 10^−2^	0.1 N
iii	5 × 10^−2^	−20 × 10^−2^	0.4 N
iv	7 × 10^−2^	−24 × 10^−2^	0.9 N
v	10 × 10^−2^	−32 × 10^−2^	1.8 N

**Table 2:** Distances and angle of two strain conditions of aligned PCL under tensile load as marked in Figure 10.

No.	Length at ε_t_		Angle at ε_t_		Projected Strain	
	2.5⋅10^−2^	5.8⋅10^−2^	2.5⋅10^−2^	5.8⋅10^−2^	ε_lx_	ε_ly_
	[µm]		[°]		[10^−2^]	[10^−2^]
1	Reference point					
2	99.7	101.8	348.3	350.0	2.7	−12.5
3	---	---	76.5 *	70.1 *	---	---
4	---	---	121.3 *	125.7 *	---	---
5	---	---	52.1 *	42.4 *	---	---
6	---	---	103.0	107.6 *	---	---
7	47.1	42.7	90.0	90.0	---	−9.3
8	---	---	119.7 *	125.2 *	---	---
9	---	---	54.8 *	51.2 *	---	---
10	---	---	117.9 *	117.5 *	---	---
11	---	---	55.3 *	51.2 *	---	---
12	---	---	28.5 *	18.5 *	---	---
13	90.2	81.1	353.7	351.9	−10.5	15.1
14	63.0	57.9	272.7	275.0	---	−8.3
15	115.6	118.6	6.6	4.5	3.0	−29.6
16	3.2	4.0	---	---	---	---
17	---	---	90.0 *	104.6	---	---
18	59.2	59.3	308.9	314.1	11.1	−7.6
19	---	---	44.5 *	27.0 *	---	---
20	102.4	103.1	16.6	14.29	1.8	−13.1

* angle between fibers.

## Data Availability

The data presented in this study are available on request from the corresponding author.
